# Diurnal variation in variables related to cognitive performance: a systematic review

**DOI:** 10.1007/s11325-023-02895-0

**Published:** 2023-08-17

**Authors:** Madhavi Munnilari, Tulasiram Bommasamudram, Judy Easow, David Tod, Evdokia Varamenti, Ben J. Edwards, Aishwarya Ravindrakumar, Chloe Gallagher, Samuel A. Pullinger

**Affiliations:** 1 Department of Exercise and Sports Science, Manipal College of Health Professions, Manipal Academy of Higher Education, Manipal, Karnataka 576104 India; 2 Institute for Physical Activity and Nutrition (IPAN), School of Exercise and Nutrition Sciences, Deakin University, Geelong, Australia; 3 Faculty of Health & Medicine, Lancaster University, Lancaster, UK; 4 Sports Science Department, Aspire Academy, Doha, Qatar; 5 Research Institute for Sport and Exercise Sciences, Liverpool John Moores University, Liverpool, UK; 6 Sport Science Department, Inspire Institute of Sport, Vidyanagar, Dist, Bellary, 583275 India

**Keywords:** Time-of-day, Circadian rhythms, Diurnal variation, Cognitive performance, Review, ROB, ROBINS-I

## Abstract

**Purpose:**

The aim of this review was to assess current evidence regarding changes in cognitive function according to time-of-day (TOD) and assess the key components of research design related to manuscripts of chronobiological nature.

**Methods:**

An English-language literature search revealed 523 articles through primary database searches, and 1868 via organization searches/citation searching. The inclusion criteria were met by eleven articles which were included in the review. The inclusion criteria set were healthy adult males, a minimum of two timepoints including morning and evening, cognitive measures of performance, and peer-reviewed academic paper.

**Results:**

It was established that cognitive performance varies with TOD and the degree of difference is highly dependent on the type of cognitive task with differences ranging from 9.0 to 34.2% for reaction time, 7.3% for alertness, and 7.8 to 40.3% for attention. The type of cognitive function was a determining factor as to whether the performance was better in the morning, evening, or afternoon.

**Conclusion:**

Although some studies did not establish TOD differences, reaction time and levels of accuracy were highest in the evening. This implies that cognitive processes are complex, and existing research is contradictory. Some studies or cognitive variables did not show any measurable TOD effects, which may be due to differences in methodology, subjects involved, testing protocols, and confounding factors. No studies met all requirements related to chronobiological research, highlighting the issues around methodology. Therefore, future research must use a rigorous, approach, minimizing confounding factors that are specific to examinations of TOD.

**Supplementary Information:**

The online version contains supplementary material available at 10.1007/s11325-023-02895-0.

## Introduction

Most of the recent research displays diurnal variation patterns in physiological and physical measures of performance when conducted in healthy adolescent males in a temperate environment (17–22 °C; [1, 2]). It is well established that repeated-sprint performances peak between 17:00 and 19:00 h with TOD differences ranging from 3.4 to 10.2% [3], while anaerobic performances have shown to peak between 16:00 and 19:30 h with TOD differences ranging from 1.8 to 12.3% [4], and time-trial performances peak between 14:00 and 20:00 h with TOD differences ranging from 2.0 to 12.0 % [5]. When isolated from external time cues, such as light (and darkness) and meal timing, endogenous circadian rhythmicity persists. Core body temperature rhythms and levels of cortisol and melatonin play an important role in circadian regulation through signals directed by the suprachiasmatic nucleus (body clock), located in the anterior part of the hypothalamus [6, 7]. Core body temperatures [8, 9], muscle temperatures [10, 11], and cortisol levels [12] peak mid-afternoon and/or early evening, while melatonin levels are at their lowest [13, 14]. The causal link of these rhythms is believed to have some implications in diurnal variation observed in human performance, whether directly or indirectly [8].

Similarly, performance variables related to cognitive abilities have also shown to fluctuate during the day [15, 16], with different variables peaking at different time-points. Timing of the peak can be explained by the multifactorial components of the cognitive task and the broad definition of cognitive performance used in the literature. The majority of studies have found simple reaction times to auditory and visual stimuli to peak in the early evening between 16:00 and 17:00 h compared to other timepoints during the day [17–20]. However, two studies have found simple reaction time scores performed in male handball goalkeepers to be best during the morning compared to other timepoints [21, 22], while it has also been found that no differences are present during the day [23]. Other cognitive performance tests related to accuracy and consistency in racquet sports serves and alertness have been found to differ in phase with core and peak body temperatures, peaking in the early afternoon or evening [24–26]. However, tasks that require fine motor control skills have been observed to peak at opposite times, with the highest values observed in the morning. Lower values are observed in the evening when negative effects are associated with an accumulation of time awake since the last sleep and low levels of arousal are present [16, 27]. Similarly, tasks related to mental arithmetic and short-term memory are also peaking in the early morning hours, highlighting that the time of peak performance is influenced by the type of the task [16].

Considering cognitive performance is multifactorial and includes many different components related to attention, accuracy, consistency, reaction time, vigilance, decision-making, and executive functions, a comprehensive review of the topic area is required to identify the gaps currently present within the literature and increase understanding within this area. It has been established that several factors related to chronobiological research design negatively influence observed findings, such as sleep, food intake, counterbalancing/randomization, and room lighting. Therefore, a standard approach to methodologies in research design while reporting research design aspects would help reduce the signal-to-noise error and ensure findings are not affected. Highlighting these potential methodological concerns and other findings related to issues around study set-up will help improve future studies. In addition, other methodological problems are present, specifically concerning the menstrual cycle definition and hormonal state. Large differences in findings related to cognitive performance are observed during different stages of the menstrual cycle.

Therefore, due to the complexity of menstrual cycles and the lack of standardization in the literature around this given area, the present manuscript aimed to assess the following research question: “In healthy adult males, what is the magnitude of diurnal (morning session vs. evening session) differences in performance variables related to cognitive performance?” Additional in-depth information related to research design deemed specifically important for chronobiological (TOD) studies will be provided to ensure future studies are more rigorous and factors can be controlled.

## Methods

### Reporting standard

This systematic review adheres to the guidelines of Preferred Reporting Items for Systematic Reviews and Meta-Analyses 2020 (PRISMA 2020) [27]. The corresponding checklist for PRISMA 2020 is provided in Appendix 1, indicating the page references for the information included in the present review.

### Eligibility criteria

The criteria for study inclusion were derived from the Cochrane guidelines for conducting systematic reviews [28]. These inclusion and exclusion criteria were established and unanimously agreed upon by all nine authors. After the initial screening of studies, three authors (AR, MM, and TB) independently evaluated the eligibility of each manuscript by examining the titles and abstracts in a standardized manner, ensuring blinding during the assessment process. To be deemed eligible, the manuscript had to meet the specified inclusion criteria:

1. Population: healthy adult male participants (18+ years of age) only (exclusion of female participants so that menstrual implications did not need to be addressed). Due to the impact of hormonal fluctuations on cognitive performance parameters, females were excluded as current research renders it difficult to interpret findings due to standardization of protocols.
2. Time-of-day: comparison between morning vs. evening cognitive performance variables with a minimum of two time-points.
3. Cognitive performance variables: such as attention, accuracy, reaction time, vigilance, consistency, and/or alertness.
4. Design: counterbalanced and/or randomized trials.

### Literature search strategy and information sources

A systematic search for English-language literature in the grey literature was performed at Liverpool John Moores University electronic library, Manipal Academy of Higher Education electronic library, and electronic databases (PubMed, Scopus, and Web of Science) from August 2021 to May 2022, concluding on May 21, 2022. The search aimed to find pertinent content concerning cognitive performance variables and their variation throughout the day, utilizing specific search syntax with Boolean operators in titles, abstracts, and keywords of indexed documents: (“time of day” OR “time-of-day” OR “daily rhythm” OR “daily variation” OR “daily fluctuation” OR “diurnal rhythm” OR “diurnal variation” OR “diurnal fluctuation” OR “circadian rhythm” OR “circadian variation” OR “circadian fluctuation”) AND (“cogni*” OR “cognitive performance” OR “attent*” OR “attention control” OR “sustained attention control” OR “selective attention” OR “accuracy” OR “alert*” OR “decision-making” OR “decision making” OR “reaction time”) was conducted (Appendix 2). The study employed supplementary advanced search methods, including the incorporation of wildcards, truncation, and proximity searching. As part of the secondary search (conducted by MM & TB), the reference lists of all included papers were manually screened to identify any additional relevant publications. Additionally, forward reference searching was conducted by exploring citations and authors to identify potential follow-up studies. To minimize potential selection bias, one author (SP) independently carried out the search for study selection. The PRISMA 2020 flow diagram [27] was used to illustrate the flow of papers, encompassing searches of databases, registers, and other sources, throughout the study selection process.

### Study selection

The article was included if the data from male participants could independently be identified in the case of the study population being both male and female. In instances where the abstract and/or the title did not provide enough information to indicate whether the article met inclusion criteria, the article was obtained and read by a third reviewer (SP), who determined the relevance of the manuscript for the review. For articles where the primary objective was not a TOD investigation, with a minimum of two timepoints (morning and evening), the manuscript was excluded. All conference abstracts, literature reviews, and letters to the editor were not included as such studies are not critically appraisable and/or methodologically-quality-assessable.

### Data extraction

The data extraction process was carried out independently by three authors (MM, AR, and TB), with a fourth author (SP) responsible for conducting a thorough data check. The information extracted from the reviewed studies encompassed the following aspects: (1) details about the study authors and date; (2) participant information, including the number of participants and their characteristics such as age, body mass, and stature; (3) the circadian chronotype questionnaire employed to assess the participants and their corresponding scores; (4) specifics regarding the time-of-day when testing sessions occurred (e.g., morning, afternoon, evening, along with the specific time); (5) the cognitive test(s) administered during the studies; (6) the equipment utilized, including rackets, shuttles, or computers; (7) the performance variables evaluated, such as attention, reaction time, accuracy, and risk-taking behavior; (8) the significance level established with *P* values; and (9) information on % differences between testing timepoints (if available), the establishment of diurnal variation, and the mean and standard deviation values.

Various factors pertinent to research design and chronobiological studies were quantified, which included room temperature control, sleep patterns, food intake, light intensity, fitness levels, and the use of randomization and counterbalancing techniques [3–5]. Each factor was recorded with a binary response of “yes” or “no,” while fitness levels were further categorized as trained or untrained. In cases where an article did not mention or refer to a specific factor, a negative response (no) was noted.

### Quality assessment

To evaluate the risk of bias in the study, two distinct tools were employed, following the Cochrane Scientific Committee’s quality assessment recommendations. The assessment of randomized studies utilized the risk of bias (ROB) 2.0 tool, while the ROBINS-I tool was applied to evaluate non-randomized studies. Although there were some similarities in features between both tools, they were primarily focused on specific outcomes. The evaluation involved fixed sets of bias domains, enabling an overall risk of bias judgment, with scores categorized from “low” to “critical.” Manuscript quality was independently assessed by two reviewers (AR and TB), who identified discrepancies in agreement across four domains of risk of bias among the 11 studies included in the review (5.6% of cases). To resolve these discrepancies, a third reviewer (SP) was consulted. For a clear visual representation of the results, Figs. 2 and 3 display a “traffic light” plot for each domain.

## Results

### Search results

**Fig. 1:**
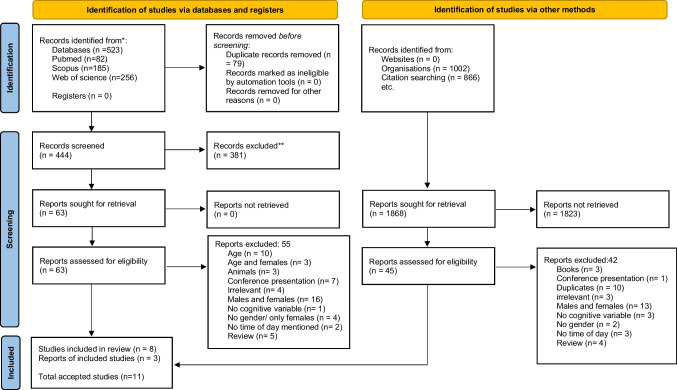
PRISMA 2020 flow diagram for new systematic reviews which included searches of databases, registers, and other sources

We initially identified 523 articles from primary database searches. Additionally, 1868 articles were found through organization searches (university databases) and citation searches. Figure 1 provides a breakdown of the number of articles found in each electronic database and other search methods, along with a comprehensive flow chart detailing the steps taken during the literature search. After eliminating duplicates, 444 titles from the databases were saved in the reference manager (Mendeley, Elsevier, Amsterdam, Netherlands). Subsequently, we thoroughly examined the titles, abstracts, and keywords of these manuscripts, resulting in 63 reports chosen for full-text analysis. Among these reports, 8 met the inclusion criteria and were included in the systematic review. Moreover, through organization searches and citation searching, we identified 45 additional reports that were evaluated for eligibility. Among these, 3 reports fulfilled the inclusion criteria and were deemed eligible, raising the total number of accepted studies to 11. Detailed explanations for exclusion can be found in Fig. 1.

### Study characteristics

Table 1 presents detailed characteristics of the participants across 11 studies, including a total of 151 male participants (with an average of 14 participants per study). The number of participants in each study ranged from 8 to 25. Among these studies, 63.6% focused on assessing circadian chronotype, with different questionnaires used, such as the morningness-eveningness questionnaire (Horne and Ostberg, 1976), the Composite Scale of Morningness [29], and a subjective amplitude scale. The results revealed that 77.1% of the participants were classified as having an intermediate chronotype, 11.0% as morning chronotype, and 11.9% as an evening chronotype. However, three studies did not report any information regarding the chronotype of their participants.

**Table 1:** Summary of the articles reviewed for cognitive performance (*n* = 11) with an overview of the participants, the experimental protocols with the time-of-day, exercise mode, performance test, the variables examined, and the main findings related to time-of-day in relation to each variable

Author & date	Participants	Chronotype assessment and distribution	Testing time-of-day	Test	Performance variables examined	Significance of main effects between condition	Main findings
Bougard et al. (2016)	20 healthy males	Morningness-eveningness questionnaire (Horne & Ostberg 1976)	*EM* = 06:00 h	Sign cancellation test (adapted from Zazzo, 1969)	Vigilance	***P*** **< 0.05**	Vigilance was significantly better in the LM and E than EM and A; 10.3% and 7.6%; *EM* = 307.3 6 ± 6.12 vs *LM* = 289.64 ± 9.33 vs *A* = 319.53 ± 6.7 vs *E* = 296.85 ± 6.67
	24.6 ± 4.6 yrs, 178.4 ± 8.9 cm, 75.7 ± 18.1 kg	20-N types	*LM* = 10:00 h	Computer-based Zimmermann & Fimm (1994) test battery	Simple reaction time	***P*** **< 0.05**	Reaction times were significantly faster in E than M and A; 9%; *EM* = 250.90 ± 6.74 vs *LM* = 242.23 ± 5.41 vs *A* = 258.53 ± 9.17 vs *E* = 237.08 ± 3.89
			*A* = 14:00 h				
			*E* = 18:00 h				
Casagrande et al. (1997)	20 male university students	NA	*M* = 11:30 h	Letter cancellation test (LCT)	LCT-2 letter-vigilance		
	21.8 ± 2.4 yrs		*EA* = 13:30 h		Hits	***P*** **< 0.05**	Hits are significantly higher in EA and N than A, LA, EE, LE
			*LA* = 15:30 h		False positives	*P* ˃ 0.05	No significant difference between any conditions
			*E* = 17:30 h		Completion time	*P* ˃ 0.05	No significant difference between any conditions
			*LE* = 19:30 h		Signal discrimination	***P*** **< 0.02**	Signal discrimination is significantly less in LA and higher in EA
			*N* = 21:30 h		Decision-making criterion	***P*** **< 0.02**	Decision-making is significantly better in LA, LE and least in EA and N
					LCT-3 letter-vigilance		
					Hits	***P*** **< 0.003**	Hits are highest in LM, A, and LE and least in N
					False positives	*P* = 0.06	No significant difference between any conditions
					Completion time	***P*** **< 0.004**	Completion time is lowest in LM and highest in LE and N
					Signal discrimination	***P*** **< 0.03**	Signal discrimination is highest in LA and lowest in LM and N
					Decision-making criterion	*P* = 0.06	No significant difference between any conditions
Ceglarek et al. (2021)	65 participants (25 males)	Chronotype questionnaire (Oginska et al. 2017)	M type—between 09:25 h to 09:55 h and between 18:30 h and 19:02 h	Signal detection theory (Green and Swets, 1966)	Accuracy	*P* = 0.372	No significant effects of time-of-day. E types were more accurate than M types both in the morning and evening session
	24.3 ± 3.6 yrs	12-M types, 13-E types	E type—between 11:00 h and 11:30 h and between 20:40 h and 21:10 h		Reaction time	***P*** **= 0.014**	Reaction times were better in the evening than morning sessions
Edwards et al. (2005)	8 male recreational badminton players	Composite scale of morningness (Smith et al. 1989)	*M* = 08:00 h	10 short and 10 long badminton serves	Serve accuracy	***P*** **= 0.039**	Serve accuracy was significantly better in A compared to M and E for long and short serves; short serve *M* = 22.2 ± 5.1 vs. *A* = 15.9 ± 2.7 vs. *E* = 19.5 ± 3.1; long serve *M* = 23.9 ± 3.9 vs. *A* = 19.7 ± 2.3 vs. *M* = 22.4 ± 6.0
	21.3 ± 2.4 yrs, 170.0 ± 2.0 cm, 69.8 ± 4.7 kg, 10.2 ± 5.4 yrs of experience	8-N types	*A* = 14:00 h		Serve consistency	*P* = 0.202	No significant difference between M, A, and E.
			*E* = 20:00 h				
Edwards et al. (2007)	12 right-handed male recreational dart players	NA	*EM* = 07:00 h	33 throws (11 blocks of 3 throws) at 2.37 m and 3.56 m from the dartboard	Accuracy	***P*** **< 0.0005**	Accuracy was better in the E > A > LM > EM in long-range throws, no change in short-range throws.
	21.4 ± 1.0 yrs, 2 yrs of experience		*LM* = 11:00 h		Consistency	***P*** **< 0.0005**	Consistency was better in the E, equal in A and LM, and least in EM in long-range throws, no change in short-range throws.
			*A* = 15:00 h				
			*E* = 19:00 h				
Hanumantha et al. (2021)	20 (10 male) undergraduate medical students	NA	*M* = 10:00 h	Simple reaction time task (PEBL version 2.0 software)	Simple reaction time	*P* = 0.741	No significant effect of time of day on simple reaction time
	Age range: 18–25 yrs		*A* = 13:00 h				
			*E* = 17:00 h				
Higuchi et al. (2000)	9 diurnally active healthy male subjects	Japanese version of the morningness-eveningness questionnaire of Horne and Östberg (Motohashi 1988)	*M* = 08:00 h	P300 test	Reaction time	*P* ˃ 0.05	No significant effect of time of day on reaction time
	29.7 ± 8.1 yrs	9-N types	*LM* = 11:00 h		Attention	***P*** **< 0.05**	Attention was significantly better at LM, A, EE, and LE than at M
			*A* = 14:00 h				
			*EE* = 17:00 h				
			*LE* = 20:00 h				
Jarraya et al. (2014a)	12 male handball goal keepers	Horne and Ösberg self-assessment questionnaire (Horne & Östberg 1976)	*M* = 08:00 h	Reaction time task (as per Jarraya et al. 2012, 2013)	Reaction time	***P*** **< 0.05**	Reaction time was better in the M than in A, EE, LE, and MN.
	18.5 ± 1.7 yrs, 1.80 ± 5.8 cm, 79 ± 4.2 kg, 8.3 ± 2.4 yrs of experience	12-N types	*A* = 12:00 h	Selective attention task (as per Jarraya et al. 2012, 2013)	Selective attention	***P*** **< 0.05**	Selective attention was better in the M than in A, EE, LE, and MN.
			*EE* = 16:00 h	Constant attention task (as per Jarraya et al. 2012, 2013)	Constant attention	***P*** **< 0.05**	Constant attention was better in the M than in A, EE, LE, and MN.
			*LE* = 20:00 h				
			*MN* = Midnight				
Jarraya et al. (2014b)	12 male handball goal keepers	Horne and Ösberg self-assessment questionnaire (Horne & Östberg 1976)	*M* = 8:00 h	The simple RT test (as per Jarraya et al. 2012)	Reaction time	***P*** **< 0.001**	Reaction time was better in the M than A, E, and MN; amplitude of 34.1 ± 4.1%
	18.5 ± 1.7 yrs, 1.80 ± 5.8 cm, 79 ± 4.2 kg, 8.3 ± 2.4 yrs of experience	12-N types	*A* = 12:00 h	Selective attention task (as per Jarraya et al. 2012)	Selective attention	***P*** **< 0.001**	Selective attention was higher in the M than LE and MN; amplitude of 40.3 ± 9.3%
			*E* = 16:00 h	Constant attention task (as per Jarraya et al. 2012)	Constant attention	**P < 0.001**	Constant attention was higher in the M than LE and MN; amplitude of 40.3 ± 9.3%
			*LE* = 20:00 h				
			*MN* = Midnight				
Reilly et al. (2007)	8 male football players	Horne and Ösberg self-assessment questionnaire (Horne & Östberg 1976)	*M* = 08:00 h	Response to a visual light stimulus	Simple reaction time	***P*** **< 0.05**	Reaction time was better in the EE than LE, M, and A; 13.4%; *M* = 365 + 65, *A* = 430 + 107, *EE* = 322 + 90, *LE* = 382 + 54
	19.1 + 1.9 yrs, 178 + 4 cm, 75.9 + 7.9 kg, 10.8 + 2.1 yrs of experience	8-N types	*A* = 12:00 h	Visual analogue scale from 0 to 10	Alertness	***P*** **< 0.001**	Alertness was found to be better in the LE than EE, M, and A; 7.3%; *M* =4.4 + 1.5, *A* = 6.1 + 1.4, *EE* = 6.8 + 1.0, *LE* = 7.3 + 1.5
			*EE* = 16:00 h				
			*LE* = 20:00 h				
Souissi et al. (2019)	15 healthy male physical education students	Horne and Östberg self-assessment questionnaire (Horne & Östberg 1976)	*EM* = 7:00 h	Reaction test	Reaction time	***P*** **< 0.05**	Reaction time was significantly better at LM and E than EM, M, A, and LA; amplitude of 10.2%; *EM* = 0.41 ± 0.02, *M* = 0.39 ± 0.02, *LM* = 0.37 ± 0.02, *A* = 0.41 ± 0.02, *LA* = 0.39 ± 0.02, *E* = 0.37 ± 0.03
	20 ± 1 yrs, 174.3 ± 4.3 cm, 70.8 ± 3.5 kg	15-N types	*M* = 09:00 h	Number cancellation test	Attention	***P*** **< 0.05**	Attention was significantly better at LM and E than EM, M, A, and LA; amplitude of 7.8%; *EM* = 66.13 ± 2.89, *M* = 68.43 ± 2.98, *LM* = 71.48 ± 3.52, *A* = 65.88 ± 2.94, *LA* = 68.55 ± 2.99, *E* = 71.45 ± 3.56
			*LM* = 11:00 h				
			*A* = 13:00 h				
			*LA* = 15:00 h				
			*E* = 17:00 h				

*M*, morning; *EM*, early morning; *LM*, late morning; *A*, afternoon; *EA*, early afternoon; *LA*, late afternoon; *E*, evening; *EE*, early evening; *LE*, late evening; *MN*, midnight; *M* type, morning type; *N* type, neither type; *E* type, evening type; *h*, hours; *Kg*, kilograms; *cm*, centimeters; *m*, meters; *yrs*, years; *n*/*a*, not available

Statistical significance (*P* < 0.05) is indicated in bold

Morning sessions took place between 06:00 and 11:30 h, while evening sessions ranged from 16:00 to 21:10 h. In addition to these time-points, ten studies used extra timepoints for assessing diurnal variation. The number of time points assessed varied, meeting the inclusion criteria of at least two time-points. Among the cognitive aspects studied, reaction time was evaluated in 8 studies (72.7%), attention in 4 studies (36.3%), accuracy in 3 studies (27.2%), consistency in 2 studies (18.1%), vigilance in 2 studies (18.1%), and alertness in 1 study (9%). Various cognitive tests were utilized, including simple reaction time tasks, letter or sign cancellation tasks, signal detection tasks, badminton serves, dart throws, p300 tests, and selective and constant attention tasks.

In ten studies, performance variables exhibited TOD effects, with significant differences between morning and evening values. Four studies reported significantly better reaction times in the evening (ranging from 9.0 to 13.4%), while two studies found better reaction times in the morning (up to 34.2%). Two other studies found no differences in reaction times across different times of the day. Attention levels were found to be lowest in the morning in two studies (7.8% amplitude) and highest in the morning in two other studies (40.3% amplitude). Accuracy levels showed some variation, with one study reporting the highest values in the afternoon (14:00 h), another in the evening, and one observing no differences. The study that assessed consistency found better values in the evening, while the other study found no differences. Alertness also displayed diurnal variation, with the highest values observed in the late evening (20:00 h) compared to morning, afternoon, or early evening values by 7.3%.

Due to significant methodological and clinical heterogeneity among the studies, a meta-analysis was not feasible. Factors such as missing data, population differences, metrics, outcomes, and study designs made it impractical to pool the data for a meta-analysis. Moreover, the relatively low number of studies (11) with small average sample sizes and high heterogeneity would likely lead to underpowered results and challenges in detecting significant effects. Consequently, the study presented unweighted results and did not pursue a meta-analysis, considering the potential for compounded errors and inappropriate summaries.

### Quality of work

Table 2 presents comprehensive information concerning various aspects of research design such as randomization, counterbalancing, light intensity recording, meal control, room temperature control, and sleep and fitness regulation. These factors are particularly crucial in conducting chronobiological investigations.

**Table 2:** Detailed information factors that specifically relate to chronobiology (time-of-day), randomization, counterbalancing, control of light intensity record, room temperature control, fitness level, and control of meals all accepted articles

Date	Author	Randomization	Counterbalancing	Record of light intensity	Control of meals	Control of room temperature	Control of sleep	Fitness
2016	Bougard et al.	Yes	Yes	No	Yes	Yes	Yes	Healthy male
2010	Casagrande et al.	No	No	No	No	No	Yes	Healthy university students
2021	Ceglarek et al.	Yes	Yes	No	Yes	No	Yes	Healthy
2005	Edwards et al.	Yes	Yes	No	Yes	No	Yes	Recreational badminton players
2007	Edwards et al.	Yes	Yes	Yes	Yes	Yes	Yes	Recreational dart players
2021	Hanumantha et al.	No	No	No	Yes	No	No	Healthy
2000	Higuchi et al.	Yes	No	No	No	No	Yes	Active healthy
2014a	Jarraya et al.	Yes	No	No	Yes	Yes	Yes	Handball goalkeepers
2014b	Jarraya et al.	Yes	No	No	No	Yes	No	Handball goalkeepers
2007	Reilly et al.	No	Yes	No	Yes	No	Yes	Football players
2019	Souissi et al.	Yes	No	No	Yes	Yes	No	Healthy male
		**8/11 = yes (73%)**	**5/11 = yes (45%)**	**1/11 = yes (9%)**	**8/11 = yes (73%)**	**5/11 =yes (45%)**	**8/11 = yes (73%)**	

It was observed that none of the studies fulfilled all the essential criteria for chronobiological research. Among the included studies, 5 of them implemented counterbalancing to minimize learning effects, while 8 studies conducted TOD sessions in a randomized order. Notably, 4 studies incorporated both counterbalancing and randomization in their protocols, while only one study lacked both. Regarding specific controls, the majority of the studies (*N* = 8) regulated meals and sleep, whereas less than half (*N* = 5) provided details about room temperature control. Remarkably, only 3 studies effectively controlled all three aspects. All 11 studies did, however, furnish information about the “fitness” levels of their participants, who were either healthy males or sports players.

### Methodological quality control and publication bias

**Fig. 2:**
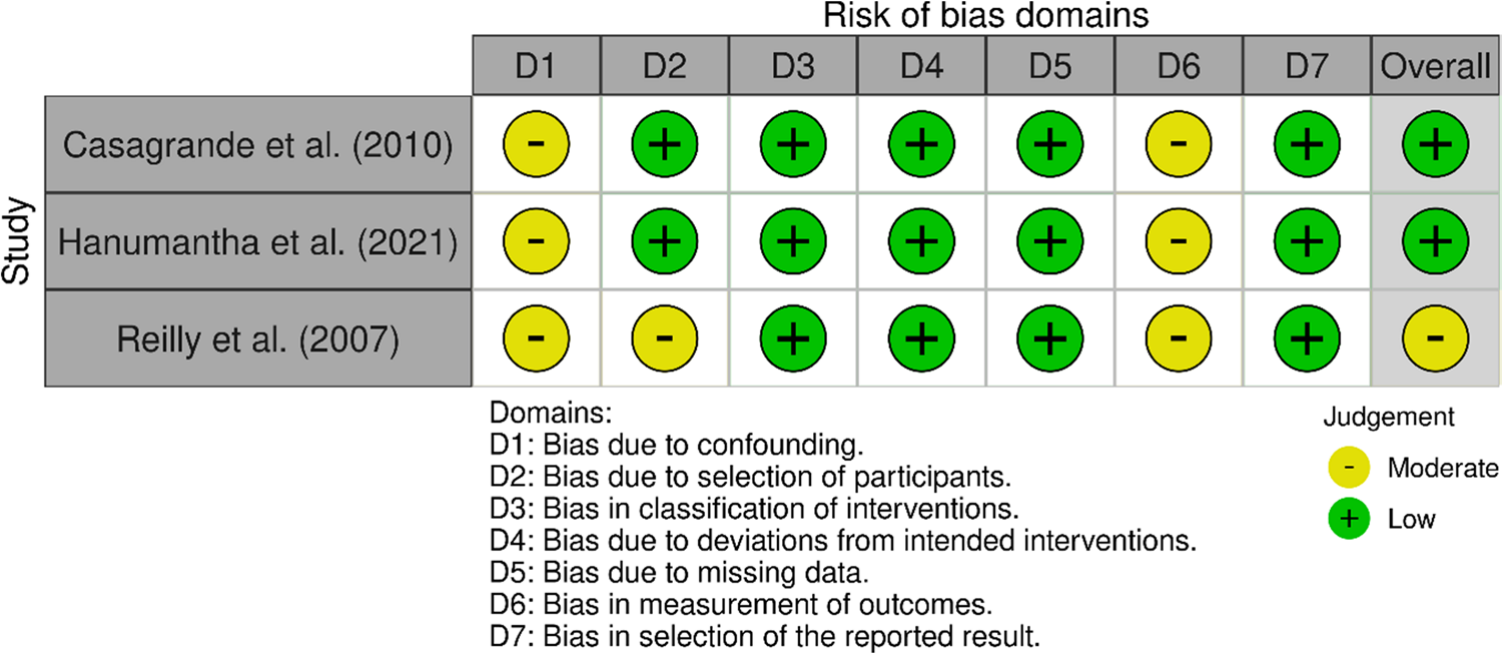
Risk of bias of the three included studies, according to the ROBINS-I tool using the “traffic light” plots of the domain-level judgements for each individual result (McGuinnes & Higgins, 2020)

Three non-randomized studies utilized the ROBINS-I tool (refer to Fig. 2), and the detailed findings are available in the same figure. All three studies exhibited a low risk of bias in the classification of interventions (domain 3) and deviations from intended interventions (domain 4). Additionally, they had a low risk of bias due to missing data (domain 5) and in the selection of reported results (domain 7). The level of bias associated with participant selection ranged from low to moderate (domain 2), while bias arising from confounding (domain 1) and bias in outcome measurement showed moderate risk (domain 6). In conclusion, two of the studies received a low overall risk of bias judgment, while one study obtained a moderate overall risk of bias judgment.

**Fig. 3:**
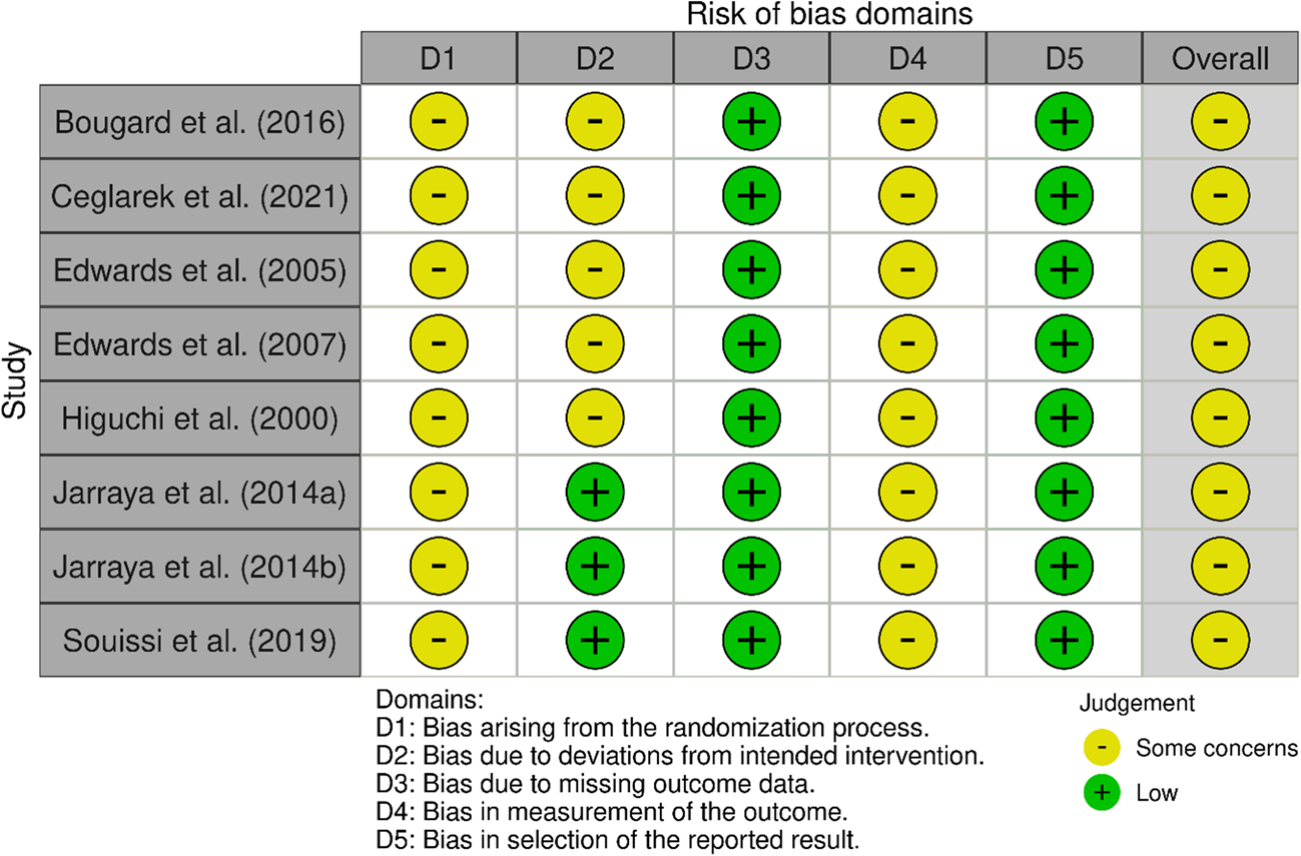
Risk of bias of the eight included studies, according to the RoB 2.0 tool using the “traffic light” plots of the domain-level judgements for each individual result (McGuinnes & Higgins, 2020)

A total of eight studies employed the risk of bias (ROB) 2.0 tool (see Fig. 3). Across all studies, there was a low risk of bias due to missing outcome data (domain 3) and the selection of reported results (domain 5). Regarding deviations from intended interventions (domain 2), the risk of bias ranged from low to moderate, while the randomization process (domain 1) and the measurement of outcomes (domain 4) showed some concerns regarding bias. In summary, all eight studies exhibited some concerns regarding the risk of bias across all domains.

## Discussion

In this recent analysis, data from 11 studies were examined to compare the diurnal variation in cognitive performance measures and assess the strength of the evidence supporting the existence of a “peak” time for cognitive functioning. The key results of this review can be summarized as follows: firstly, a significant majority of the papers (90.9%, *N* = 10) revealed variations in cognitive performance related to the TOD for at least one cognitive performance variable. Secondly, the TOD peak for cognitive performance varied depending on the specific cognitive variable under assessment. Lastly, certain limitations and concerns were identified, particularly regarding the methodology, study control and overall quality of the included studies.

### Cognitive performance

Four studies have investigated the TOD effects on attention (Table 1) [20, 22, 30, 31]. Two studies reported better selective attention and constant attention in the morning (08:00 h) using a selective attention test, with values declining as the day progressed potentially due to the training experience of the players recruited in the various studies [21, 22]. Interactions around daytime sleepiness, time awake, and sleep build-up influence TOD aspects related to cognitive function [32, 33], thus suggesting that observations around attention are highly affected by sleep homeostasis. However, a study performed by Higuchi et al. (2000) reported reduced attention levels in the morning (08:00 h) compared to the late morning (11:00 h) which was sustained until late evening (20:00 h) using a P300 test. Another study performed by Souissi et al. (2019) reported reduced attention levels during the early morning (07:00 h), morning (09:00 h), afternoon (13:00 h), and late afternoon (15:00 h) compared to the afternoon (11:00 h) and evening (17:00 h) when using a number cancellation test. Overall, cognitive performance related to attention displayed contradictory findings; however, these variations can be attributed to the fact that different tests were used to assess attention, such as a P300 test, a selective attention test, and a number cancellation test, thus making it difficult to compare findings between different journals. It is well established that sleep inertia is affected by the circadian phase, and when subjective ratings of fatigue values are higher, visual and/or selective attention performance is negatively affected in the morning. In addition, in the post-lunch dip, sleepiness has been found to increase and attention has been found to decrease, with reduced alertness, subjective sleepiness, fatigue, and negative mood states increased [31, 34].

Two studies investigated TOD effects on vigilance, which varied with the outcome assessment type. When using an adapted sign cancellation test, vigilance is reported to be better in the late morning (10:00 h) and evening (18:00 h) than in the early morning (06:00 h) and afternoon (14:00 h) [35]. The observed variations in vigilance might be due to the improvement in visuomotor coordination [36] and core temperature [37]. The increase in motor contractile properties [38] during the day might be responsible for the increase in motor coordination and an increase in nerve conduction velocity [39] which in turn leads to better visuomotor coordination. When the letter cancellation test (LCT) is used, the LCT of 2 letters demonstrates several variables to display significant variation over TOD, with peaks occurring at different times of the day based on the performance variable examined. Similarly, an LCT of 3 letters demonstrated findings in-line with the observations present in the LCT of 2 letters [40].

Eight studies investigated TOD effects on reaction time (Table 1). Four studies demonstrated a faster reaction time in the evening which ranged from 9 to 13.4% when compared to other times of the day [17, 18, 20, 25]. In fact, late morning (10:00 h) values also showed better reaction times than morning, and afternoon values [20, 37]. Two studies reported faster reaction times in the morning than other times of the day when using a simple reaction time test, finding reaction times to reduce as the day progressed, with an amplitude of 34.1% [21, 22]. There was a decline in the reaction time performance post midday in comparison to morning, which might be due to the accumulation of tiredness after midday [21, 22]. These results are in line with a study that reported a fall in performance in the afternoon due to tiredness resulting from time awake [25]. The discrepancies between studies in the literature could be due to the training experience of the participants and due to differences in the population (trained vs untrained) recruited in the studies [22]. It has previously been established that amplitude of TOD differences between morning and evening is higher in trained compared to untrained individuals [41]. Furthermore, the level of training can also affect the reaction time performances, as suggested by a previous study which found that people who exercised regularly had faster reaction times compared to sedentary people [42]. Interestingly, two studies did not show any significant diurnal variation in reaction time [23, 43], due to the complexity of the tasks [23]. Nevertheless, time of the day and duration of time awake play a major role in response time observed [23] as reported by an earlier study that showed an increase in wakeful time and adverse circadian phases resulted in a delayed reaction time [44].

One study investigated the TOD effect on alertness and observed that alertness peaks in the late evening (20:00 h) by up to 7.3 % and was lowest in the morning (08:00 h). It is believed that the increase in body temperature might lead to physiological arousal that enhances cognitive performance as it modulates neurobehavioral performance [45]. Three studies investigated the effect of TOD on accuracy with discrepancies in the results present in all three studies. The study performed by Edwards et al. (2005) found better badminton serve accuracy values in the afternoon (14:00 h) compared to morning (08:00 h) and evening (20:00 h) in both short and long serves. These findings are like the findings observed in tennis serves, where both accuracy tests displayed high levels of test-retest reliability [24]. Another study found dart-throwing accuracy to significantly improve as the day progressed with the best accuracy observed in the evening (19:00 h) compared to the afternoon (15:00 h) and better than the morning (07:00 h) [25] in long-distance throws only. Similar observations were reported in the consistency of dart throws with the highest consistency present in the evening (19:00 h), compared to values observed in the early morning (07:00 h). As dart throwing requires a combination of hand-eye coordination and muscle contraction, when performing longer dart throws there is a larger emphasis placed on muscle contraction (strength), thus, findings established have observed TOD variations in line with core body temperature [26, 41]. In shorter throws, the emphasis is placed more on control mechanisms and factors related to fatigue, hence little to no variation of TOD established [15, 26]. However, one study did not show any significant difference throughout the day in accuracy for hits, false alarms, correct rejections and misses [18]. However, chronotype of the individual was found to affect accuracy, with evening types being more accurate than morning types in both morning and evening sessions. Accuracy is not parallel with the circadian patterns of body temperature with lower levels of accuracy present when temperature was at its highest [25]. These results depend on the skill level of the players recruited in this study [46], the fatigue levels due to time awake [15], changes in the “basal arousal.” Other reasons for the conflicting results may be related to the variation and lack of control in factors deemed important for TOD research (Table 2).

### Methodological quality and control

As far as we are aware, only three systematic reviews have looked into issues around chronobiology study design [3–5]. In agreement with these previous reviews, an apparent lack of control in the research studies selected was also established within this review. It is well known that the periodicity of the body clock in human beings is influenced by external environmental rhythmic cues which impact the constant adjustment of the body clock (zeitgebers). In TOD studies, rhythmic cues such as activity, feeding/fasting, and light-dark cycles are the main factors that require additional control [47]. Light intensity was only reported in one study (9%), with no other studies reporting any information around light or dark exposure. The regulation of alertness and mood in human studies is highly affected by light exposure [47, 48]. Studies have observed that light exposure influences several cognitive processes related to attention, memory, and arousal [49–52], with short-wavelength light negatively affecting reaction times [53]. Although there is a lack of clarity regarding whether or not light exposure results in increased cognitive performance for cognitive tasks which require sustained attention, light exposure is believed to improve such performance [47, 48, 54]. Therefore, there is a great importance to control light and/or dark exposure in cognitive studies. Three studies (27 %) failed to provide information around the control of meals, a factor which plays a vital role in cognitive performance. It has been established that “meals” potentially improve cognition and alertness [55], with the timing of meals, the characteristics of the meal, and the timing of meals affecting cognitive performance [56]. The size and macronutrient content of the meal influence mental performance, while the TOD at which a meal is consumed will affect cognitive performance [56]. In addition, alterations in meal timings have been shown to improve cognition [55]. The lack of standardization makes the comparison of results challenging. All studies reported information related to participant “fitness levels,” although it must be noted that personal characteristics of individuals can influence cognitive performance, such as age and level of training (sedentary vs. non-sedentary), which play a major role. It has been found that the level of training is closely associated with a better brain structure and brain functioning and, thus, results in better cognitive performance in trained individuals [57, 58].

When looking at sleep, 3 studies (27 %) failed to provide any information related to maintaining similar sleeping habits to “normal life.” No information was provided to participants to ensure habitual rising and waking times were maintained, that they should not stay up late, or whether any of the individuals had a prevalence of sleep insomnia or were sleep deprived. Sleep plays a significant role in cognitive performance and lack of sleep has shown to have negative effects on an extensive variety of performance variables related to decision-making, memory and attention [59–61]. Reaction times and focused attention are worsened with one or two nights without sleep [59]. The presence of TOD in cognitive performance is highly associated with sleep homeostasis, time awake and previous sleep drive, and circadian rhythmicity, but how these processes interrelate is not well known. Diurnal variation in cognitive performance will differ in accordance with the “sleep status” of the individual. Individuals who are sleep deprived perform better around midday in tasks requiring episodic memory, while well-rested individuals showed more stable performance [60]. The time-since-last sleep is closely related to increased levels of fatigue, and as the amount of time awake increases, a negative effect is observed on cognitive performance, on restorative influences of sleep, and on arousal [62]. In addition, chronotype of individuals has also shown to play a role in simple and complex measures of cognitive performance [63]. It is well known that evening types have a significantly higher daytime sleepiness, thus resulting in worse cognitive performance in the morning when compared to morning types [16]. A total of 3 studies (27%) failed to assess chronotype scores in their participants.

Finally, other important factors to report are the mean ± SD of familiarization sessions. Not providing detailed and accurate statistics and information around this displays random and systemic bias. When counterbalancing and randomizing sessions, internal validity is guaranteed through the control of potential confounders. These are created by the effects of sequence and order and remove selection bias, balance, and both known and unknown confounding factors. In this systematic review, seven of the studies (64%) randomized their sessions, and only three (27%) counterbalanced their sessions. Significant methodological differences were observed across the accepted research manuscripts with the amount and type of familiarization varying across studies. Appropriate familiarization will ensure cognitive performance prior to conducting experimental sessions, which demonstrates a plateau effect [3].

As previously suggested in TOD studies, the importance of establishing laboratory-based protocols which are more rigorous is essential. There is a need of the methodological control and quality to improve, such as the appropriate timing of morning and evening sessions when assessing cognitive performance. The timing of morning and evening sessions assessing cognitive performance varied from 06:00 to 11:30 h and between 16:00 and 21:10 h, which is not within the appropriate timeframe needed to establish diurnal variation with timings closer to the nadir and peak of body temperature deemed more suitable. The lack of standardization of methodologies and factors that affect cognitive performance might explain why findings observed conflicting differences in several performance variables. The willingness of individuals to undertake early morning sessions and the laboratory opening times within research “buildings” affect this.

### Strength and weaknesses

One of the major strengths of this systematic review is that it is the first review providing an in-depth overview related to cognitive performance and TOD. The review was performed in a structured manner following the PRISMA 2020 guidelines [27]. In addition, only four other reviews have provided detailed information around factors affecting cognitive performance in chronobiological studies [3–5]. Further, the diversity and range of databases used within this review’s search strategy and the specific search terms utilized further strength. Finally, the inclusion criteria were strongly adhered to, and only studies which assessed diurnal variation and cognitive performance were included.

A limitation of the present systematic review was the large differences in methodology and cognitive performance tests used in the 11 included studies. Hence we were unable to conduct a meta-analysis and pool the datasets observed to further assess evidence associated to cognitive performance and TOD. This was mainly due to differences present between the 11 studies related to methodological and clinical heterogeneity [64]. Study design across studies displayed irregularities when assessing the methodological design. There was also disagreement as to whether cognitive performance displays TOD or diurnal variation and the timing of when this was observed.

## Conclusion

The present systematic review confirms that variation in cognitive performance is TOD and variable dependant. Some of the observed variations can potentially be explained by differences in body and core temperatures in the morning compared to the evening. However, more recent studies suggest that TOD variations in cognitive processes are more complex. Some of the reasons as to why some studies or variables do not display any significant TOD effects are related to differences in testing methodologies, the participants included, and confounding factors. Therefore, factors related to chronobiological research studies need to be controlled effectively. Timing tests as closely as possible to timepoints of the rhythm of core body temperature is required in future studies.

### Supplementary information

ESM 1 (DOCX 32 kb)

ESM 2 (DOCX 33 kb)

## Data Availability

My manuscript has no associated data.

## Abbreviations

LCT: Letter cancellation test
ROB: Risk of bias
TOD: Time of day
