# Supplementary material for: Diurnal variation in variables related to cognitive performance: a systematic review
Source: Sleep Breath. 2023 Aug 17;28(1):495–510. doi: 10.1007/s11325-023-02895-0 (PMC10955027; doi:10.1007/s11325-023-02895-0)
Supplement: Supplementary file 2 — (DOCX 33 kb) [file 11325_2023_2895_MOESM2_ESM.docx]

**Appendix 2. Literature search strategy example for PubMed (MEDLINE)**

**Search Syntax**

(“time of day” OR “time-of-day” OR “daily rhythm” OR “daily variation” OR “daily fluctuation” OR “diurnal rhythm” OR “diurnal variation” OR “diurnal fluctuation” OR “circadian rhythm” OR “circadian variation” OR “circadian fluctuation”)

AND

(“cogni*” OR “cognitive performance” OR “attent*” OR “attention control” OR “sustained attention control” OR “selective attention” OR “accuracy” OR “alert*” OR “decision-making” OR “decision making” OR “reaction time”)

**Records identified and screened**

**N = 82**

1. Trinh P, Hoover DR, Sonnenberg FA. Time-of-day changes in physician clinical decision making: A retrospective study. PLoS One. 2021 Sep. 17;16(9):e0257500. doi: 10.1371/journal.pone.0257500. PMID: 34534247; PMCID: PMC8448311.

2. Khemila S, Abedelmalek S, Romdhani M, Souissi A, Chtourou H, Souissi N. Listening to motivational music during warming-up attenuates the negative effects of partial sleep deprivation on cognitive and short-term maximal performance: Effect of time of day. Chronobiol Int. 2021 Jul;38(7):1052-1063. doi: 10.1080/07420528.2021.1904971. Epub 2021 Apr 19. PMID: 33874838.

3. Sharma A, Mohammad A, Saini AK, Goyal R. Neuroprotective Effects of Fluoxetine on Molecular Markers of Circadian Rhythm, Cognitive Deficits, Oxidative Damage, and Biomarkers of Alzheimer's Disease-Like Pathology Induced under Chronic Constant Light Regime in Wistar Rats. ACS Chem Neurosci. 2021 Jun 16;12(12):2233-2246. doi: 10.1021/acschemneuro.1c00238. Epub 2021 May 24. PMID: 34029460.

4. Wilkins D, Tong X, Leung MHY, Mason CE, Lee PKH. Diurnal variation in the human skin microbiome affects accuracy of forensic microbiome matching. Microbiome. 2021 Jun 5;9(1):129. doi: 10.1186/s40168-021-01082-1. PMID: 34090519; PMCID: PMC8180031.

5. van Andel E, Bijlenga D, Vogel SWN, Beekman ATF, Kooij JJS. Effects of chronotherapy on circadian rhythm and ADHD symptoms in adults with attention-deficit/hyperactivity disorder and delayed sleep phase syndrome: a randomized clinical trial. Chronobiol Int. 2021 Feb;38(2):260-269. doi: 10.1080/07420528.2020.1835943. Epub 2020 Oct 29. PMID: 33121289.

6. S H, Kamath A, Shastry R. Diurnal Variation in Visual Simple Reaction Time between and within Genders in Young Adults: An Exploratory, Comparative, Pilot Study. ScientificWorldJournal. 2021 Jan 22;2021:6695532. doi: 10.1155/2021/6695532. PMID: 33551687; PMCID: PMC7846399.

7. Pillai JA, Bena J, Bekris LM, Foldvary-Schaefer N, Heinzinger C, Rao S, Rao SM, Leverenz JB, Mehra R. Unique Sleep and Circadian Rhythm Dysfunction Neuroinflammatory and Immune Profiles in Alzheimer's Disease with Mild Cognitive Impairment. J Alzheimers Dis. 2021;81(2):487-492. doi: 10.3233/JAD-201573. PMID: 33814445; PMCID: PMC8179975.

8. Li M, Mai Z, Yang J, Zhang B, Ma N. Ideal Time of Day for Risky Decision Making: Evidence from the Balloon Analogue Risk Task. Nat Sci Sleep. 2020 Jul 16;12:477-486. doi: 10.2147/NSS.S260321. PMID: 32765144; PMCID: PMC7381795.

9. Burgess JL, Bradley AJ, Anderson KN, Gallagher P, McAllister-Williams RH. The relationship between physical activity, BMI, circadian rhythm, and sleep with cognition in bipolar disorder. Psychol Med. 2020 Jun 29:1-9. doi: 10.1017/S003329172000210X. Epub ahead of print. PMID: 32597742.

10. Zhang Y, Wang W, Li Y, Shen J, Zhang T. Does circadian rhythm disruption during their early development have lasting effects on cognition of the elder rats? Neuroreport. 2020 May 7;31(7):544-550. doi: 10.1097/WNR.0000000000001443. PMID: 32282585.

11. He Y, Li Y, Zhou F, Qi J, Wu M. Decreased circadian fluctuation in cognitive behaviors and synaptic plasticity in APP/PS1 transgenic mice. Metab Brain Dis. 2020 Feb;35(2):343-352. doi: 10.1007/s11011-019-00531-z. Epub 2019 Dec 26. PMID: 31879834

12. Takahashi T, Haitani T, Tanaka F, Yamagishi T, Kawakami Y, Shibata S, Kumano H. Effects of the time-of-day (morning vs. afternoon) of implementing a combined physical and cognitive exercise program on cognitive functions and mood of older adults: A randomized controlled study. Adv Gerontol. 2020;33(3):595-599. PMID:33280348

13. Fiala M, Lau YCC, Aghajani A, Bhargava S, Aminpour E, Kaczor-Urbanowicz KE, Mirzoyan H, Nichols I, Ko MW, Morselli M, Santana J, Dang J, Sayre J, Paul K, Pellegrini M. Omega-3 Fatty Acids Increase Amyloid-β Immunity, Energy, and Circadian Rhythm for Cognitive Protection of Alzheimer's Disease Patients Beyond Cholinesterase Inhibitors. J Alzheimers Dis. 2020;75(3):993-1002. doi: 10.3233/JAD-200252. PMID: 32390637.

14. Chan YC, Wu CS, Wu TC, Lin YH, Chang SJ. A Standardized Extract of Asparagus officinalis Stem (ETAS) Ameliorates Cognitive Impairment, Inhibits Amyloid β Deposition via BACE-1 and Normalizes Circadian Rhythm Signaling via MT1 and MT2. Nutrients. 2019 Jul 17;11(7):1631. doi: 10.3390/nu11071631. PMID: 31319549; PMCID: PMC6683278.

15. Paganini-Hill A, Bryant N, Corrada MM, Greenia DE, Fletcher E, Singh B, Floriolli D, Kawas CH, Fisher MJ. Blood Pressure Circadian Variation, Cognition and Brain Imaging in 90+ Year-Olds. Front Aging Neurosci. 2019 Apr 17;11:54. doi: 10.3389/fnagi.2019.00054. PMID: 31057391; PMCID: PMC6478755.

16. Souissi Y, Souissi M, Chtourou H. Effects of caffeine ingestion on the diurnal variation of cognitive and repeated high-intensity performances. Pharmacol Biochem Behav. 2019 Feb;177:69-74. doi: 10.1016/j.pbb.2019.01.001. Epub 2019 Jan 3. PMID: 30611752.

17. Lunsford-Avery JR, Kollins SH. Editorial Perspective: Delayed circadian rhythm phase: a cause of late-onset attention-deficit/hyperactivity disorder among adolescents? J Child Psychol Psychiatry. 2018 Dec;59(12):1248-1251. doi:10.1111/jcpp.12956. Epub 2018 Sep 3. PMID: 30176050; PMCID: PMC6487490.

18. Thomas P, He F, Mazumdar S, Wood J, Bhatia T, Gur RC, Gur RE, Buysse D, Nimgaonkar VL, Deshpande SN. Joint analysis of cognitive and circadian variation in Schizophrenia and Bipolar I Disorder. Asian J Psychiatr. 2018 Dec;38:96-101. doi: 10.1016/j.ajp.2017.11.006. Epub 2017 Nov 7. PMID: 29158147; PMCID: PMC5938152.

19. Nowack K, Van Der Meer E. The synchrony effect revisited: chronotype, time of day and cognitive performance in a semantic analogy task. Chronobiol Int. 2018 Nov;35(12):1647-1662. doi: 10.1080/07420528.2018.1500477. Epub 2018 Aug 7. PMID: 30085831.

20. Wildi K, Singeisen H, Twerenbold R, Badertscher P, Wussler D, Klinkenberg LJJ, Meex SJR, Nestelberger T, Boeddinghaus J, Miró Ò, Martin-Sanchez FJ, Morawiec B, Muzyk P, Parenica J, Keller DI, Geigy N, Potlukova E, Sabti Z, Kozhuharov N, Puelacher C, du Fay de Lavallaz J, Rubini Gimenez M, Shrestha S, Marzano G, Rentsch K, Osswald S, Reichlin T, Mueller C; APACE Investigators. Circadian rhythm of cardiac troponin I and its clinical impact on the diagnostic accuracy for acute myocardial infarction. Int J Cardiol. 2018 Nov 1;270:14-20. doi: 10.1016/j.ijcard.2018.05.136. Epub 2018 Jun 4. PMID: 29891238.

21. Facer-Childs ER, Boiling S, Balanos GM. The effects of time of day and chronotype on cognitive and physical performance in healthy volunteers. Sports Med Open. 2018 Oct 24;4(1):47. doi: 10.1186/s40798-018-0162-z. PMID: 30357501; PMCID: PMC6200828.

22. Hurdiel R, Riedy SM, Millet GP, Mauvieux B, Pezé T, Elsworth-Edelsten C, Martin D, Zunquin G, Dupont G. Cognitive performance and self-reported sleepiness are modulated by time-of-day during a mountain ultramarathon. Res Sports Med. 2018 Oct-Dec;26(4):482-489. doi: 10.1080/15438627.2018.1492401. Epub 2018 Jul 4. PMID: 29973086.

23. Song J, Chu S, Cui Y, Qian Y, Li X, Xu F, Shao X, Ma Z, Xia T, Gu X. Circadian rhythm resynchronization improved isoflurane-induced cognitive dysfunction in aged mice. Exp Neurol. 2018 Aug;306:45-54. doi: 10.1016/j.expneurol.2018.04.009. Epub 2018 Apr 13. PMID: 29660304.

24. Buchhorn R, Koenig J, Jarczok MN, Eichholz H, Willaschek C, Thayer JF, Kaess M. A case series on the potential effect of omega-3-fatty acid supplementation on 24-h heart rate variability and its circadian variation in children with attention deficit (hyperactivity) disorder. Atten Defic Hyperact Disord. 2018 Jun;10(2):135-139. doi: 10.1007/s12402-017-0240-y. Epub 2017 Oct 3. PMID: 28975530

25. Gratton C, Laumann TO, Nielsen AN, Greene DJ, Gordon EM, Gilmore AW, Nelson SM, Coalson RS, Snyder AZ, Schlaggar BL, Dosenbach NUF, Petersen SE. Functional Brain Networks Are Dominated by Stable Group and Individual Factors, Not Cognitive or Daily Variation. Neuron. 2018 Apr 18;98(2):439-452.e5. doi: 10.1016/j.neuron.2018.03.035. PMID: 29673485; PMCID: PMC5912345.

26. Logan S, Owen D, Chen S, Chen WJ, Ungvari Z, Farley J, Csiszar A, Sharpe A, Loos M, Koopmans B, Richardson A, Sonntag WE. Simultaneous assessment of cognitive function, circadian rhythm, and spontaneous activity in aging mice. Geroscience. 2018 Apr;40(2):123-137. doi: 10.1007/s11357-018-0019-x. Epub 2018 Apr 24. PMID: 29687240; PMCID: PMC5964055.

27. Diago EB, Martínez-Horta S, Lasaosa SS, Alebesque AV, Pérez-Pérez J, Kulisevsky J, Del Val JL. Circadian Rhythm, Cognition, and Mood Disorders in Huntington's Disease. J Huntingtons Dis. 2018;7(2):193-198. doi: 10.3233/JHD-180291. PMID: 29843249.

28. Qasrawi SO, Pandi-Perumal SR, BaHammam AS. The effect of intermittent fasting during Ramadan on sleep, sleepiness, cognitive function, and circadian rhythm. Sleep Breath. 2017 Sep;21(3):577-586. doi: 10.1007/s11325-017-1473-x. Epub 2017 Feb 11. PMID: 281901

29. Mazzucco S, Li L, Tuna MA, Pendlebury ST, Frost R, Wharton R, Rothwell PM; Oxford Vascular Study. Time-of-Day Could Affect Cognitive Screening Performance in Older Patients with TIA and Stroke. Cerebrovasc Dis. 2017;43(5-6):290-293. doi: 10.1159/000456673. Epub 2017 Mar 21. PMID: 28319944; PMCID: PMC5475237.

30. Correa A, Ruiz-Herrera N, Ruz M, Tonetti L, Martoni M, Fabbri M, Natale V. Economic decision-making in morning/evening-type people as a function of time of day. Chronobiol Int. 2017;34(2):139-147. doi: 10.1080/07420528.2016.1246455. Epub 2016 Oct 28. PMID: 27791397.

31. Bron TI, Bijlenga D, Kooij JJ, Vogel SW, Wynchank D, Beekman AT, Penninx BW. Attention-deficit hyperactivity disorder symptoms add risk to circadian rhythm sleep problems in depression and anxiety. J Affect Disord. 2016 Aug;200:74-81. doi: 10.1016/j.jad.2016.04.022. Epub 2016 Apr 16. PMID: 27128360.

32. Ingram KK, Ay A, Kwon SB, Woods K, Escobar S, Gordon M, Smith IH, Bearden N, Filipowicz A, Jain K. Molecular insights into chronotype and time-of-day effects on decision-making. Sci Rep. 2016 Jul 8;6:29392. doi: 10.1038/srep29392. PMID: 27388366; PMCID: PMC4937423.

33. Fimm B, Brand T, Spijkers W. Time-of-day variation of visuo-spatial attention. Br J Psychol. 2016 May;107(2):299-321. doi: 10.1111/bjop.12143. Epub 2015 Aug 7. PMID: 26248950.

34. Vogel SW, Bijlenga D, Tanke M, Bron TI, van der Heijden KB, Swaab H, Beekman AT, Kooij JJ. Circadian rhythm disruption as a link between Attention-Deficit/Hyperactivity Disorder and obesity? J Psychosom Res. 2015 Nov;79(5):443-50. doi: 10.1016/j.jpsychores.2015.10.002. Epub 2015 Oct 8. PMID: 26526321

35. Holt GB. Time of day of cognitive tests might distort shift work study results. Occup Environ Med. 2015 May;72(5):381-2. doi: 10.1136/oemed-2014-102693. Epub 2015 Mar 23. PMID: 25802288.

36. Marquié JC, Tucker P, Folkard S, Gentil C, Ansiau D. Author response to Time of day of cognitive tests might distort shift-work study results. Occup Environ Med. 2015 May;72(5):382. doi: 10.1136/oemed-2014-102788. Epub 2015 Mar . PMID: 25780029.

37. Kume Y, Sugita T, Oga K, Kagami K, Igarashi H. A pilot study: comparative research of social functioning, circadian rhythm parameters, and cognitive function among institutional inpatients, and outpatients with chronic schizophrenia and healthy elderly people. Int Psychogeriatr. 2015 Jan;27(1):135-43. doi: 10.1017/S1041610214001604. Epub 2014 Aug 5. PMID: 25092490

38. Landry GJ, Liu-Ambrose T. Buying time: a rationale for examining the use of circadian rhythm and sleep interventions to delay progression of mild cognitive impairment to Alzheimer's disease. Front Aging Neurosci. 2014 Dec 8;6:325. doi:10.3389/fnagi.2014.00325. PMID: 25538616; PMCID: PMC4259166.

39. Anderson JAE, Campbell KL, Amer T, Grady CL, Hasher L. Timing is everything:Age differences in the cognitive control network are modulated by time of day. Psychol Aging. 2014 Sep;29(3):648-657. doi: 10.1037/a0037243. Epub 2014 Jul 7. PMID: 24999661; PMCID: PMC4898963.

40. van Schie MK, Alblas EE, Thijs RD, Fronczek R, Lammers GJ, van Dijk JG. The influences of task repetition, napping, time of day, and instruction on the Sustained Attention to Response Task. J Clin Exp Neuropsychol. 2014;36(10):1055-65. doi: 10.1080/13803395.2014.968099. PMID: 25494633.

41. Hourihan KL, Benjamin AS. State-based metacognition: how time of day affects the accuracy of metamemory. Memory. 2014;22(5):553-8. doi: 10.1080/09658211.2013.804091. Epub 2013 Jun 6. PMID: 23742008; PMCID: PMC3818346.

42. Kooij JJ, Bijlenga D. The circadian rhythm in adult attention- deficit/hyperactivity disorder: current state of affairs. Expert Rev Neurother. 2013 Oct;13(10):1107-16. doi: 10.1586/14737175.2013.836301. PMID: 24117273.

43. Deschamps T, Magnard J, Cornu C. Postural control as a function of time-of-day: influence of a prior strenuous running exercise or demanding sustained-attention task. J Neuroeng Rehabil. 2013 Mar 1;10:26. doi:10.1186/1743-0003-10-26. PMID: 23452958; PMCID: PMC3598559.

44. Knight M, Mather M. Look out-it's your off-peak time of day! Time of day matters more for alerting than for orienting or executive attention. Exp Aging Res. 2013;39(3):305-21. doi: 10.1080/0361073X.2013.779197. PMID: 23607399; PMCID: PMC4067093.

45. Hunt MG, Bienstock SW, Qiang JK. Effects of diurnal variation on the Test of Variables of Attention performance in young adults with attention-deficit/hyperactivity disorder. Psychol Assess. 2012 Mar;24(1):166-172. doi: 10.1037/a0025233. Epub 2011 Sep 12. PMID: 21910547.

46. Kiryk A, Mochol G, Filipkowski RK, Wawrzyniak M, Lioudyno V, Knapska E, Gorkiewicz T, Balcerzyk M, Leski S, Leuven FV, Lipp HP, Wojcik DK, Kaczmarek L. Cognitive abilities of Alzheimer's disease transgenic mice are modulated by social context and circadian rhythm. Curr Alzheimer Res. 2011 Dec;8(8):883-92. doi: 10.2174/156720511798192745. PMID: 22171952.

47. Yan TC, Dudley JA, Weir RK, Grabowska EM, Peña-Oliver Y, Ripley TL, Hunt SP, Stephens DN, Stanford SC. Performance deficits of NK1 receptor knockout mice in the 5-choice serial reaction-time task: effects of d-amphetamine, stress and time of day. PLoS One. 2011 Mar 7;6(3):e17586. doi: 10.1371/journal.pone.0017586. PMID: 21408181; PMCID: PMC3049786.

48. Kovach CR, Woods DL, Logan BR, Raff H. Diurnal variation of cortisol in people with dementia: relationship to cognition and illness burden. Am J Alzheimers Dis Other Demen. 2011 Mar;26(2):145-50. doi: 10.1177/1533317510397329. Epub 2011 Jan 27. PMID: 21273205; PMCID: PMC3060946.

49. Hunt MG, Momjian AJ, Wong KK. Effects of diurnal variation and caffeine consumption on Test of Variables of Attention (TOVA) performance in healthy young adults. Psychol Assess. 2011 Mar;23(1):226-233. doi: 10.1037/a0021401. PMID: 21244169.

50. Reid KJ, McGee-Koch LL, Zee PC. Cognition in circadian rhythm sleep disorders. Prog Brain Res. 2011;190:3-20. doi: 10.1016/B978-0-444-53817-8.00001-3. PMID: 21531242

51. van der Heijden KB, de Sonneville LM, Althaus M. Time-of-day effects on cognition in preadolescents: a trails study. Chronobiol Int. 2010 Oct;27(9-10):1870-94. doi: 10.3109/07420528.2010.516047. PMID: 20969529.

52. Madhusoodanan S, Madhusoodanan N, Serper M, Sullivan SJ, D'Antonio E, Negi R, Brenner R. Cognitive status changes based on time of day in nursing home patients with and without dementia. Am J Alzheimers Dis Other Demen. 2010 Sep;25(6):498-504. doi: 10.1177/1533317510372373. Epub 2010 Jun 17. PMID: 20558850

53. Aziz NA, Anguelova GV, Marinus J, Lammers GJ, Roos RA. Sleep and circadian rhythm alterations correlate with depression and cognitive impairment in Huntington's disease. Parkinsonism Relat Disord. 2010 Jun;16(5):345-50. doi: 10.1016/j.parkreldis.2010.02.009. Epub 2010 Mar 16. PMID: 20236854.

54. Van Veen MM, Kooij JJ, Boonstra AM, Gordijn MC, Van Someren EJ. Delayed circadian rhythm in adults with attention-deficit/hyperactivity disorder and chronic sleep-onset insomnia. Biol Psychiatry. 2010 Jun 1;67(11):1091-6. doi: 10.1016/j.biopsych.2009.12.032. Epub 2010 Feb 16. PMID: 20163790.

55. Elsheikh TM, Kirkpatrick JL, Fischer D, Herbert KD, Renshaw AA. Does the time of day or weekday affect screening accuracy? A pilot correlation study with cytotechnologist workload and abnormal rate detection using the ThinPrep Imaging System. Cancer Cytopathol. 2010 Feb 25;118(1):41-6. doi: 10.1002/cncy.20060. PMID: 20099317.

56. An M, Huang J, Shimomura Y, Katsuura T. Time-of-day-dependent effects of monochromatic light exposure on human cognitive function. J Physiol Anthropol. 2009 Sep;28(5):217-23. doi: 10.2114/jpa2.28.217. PMID: 19823003.

57. Matchock RL, Mordkoff JT. Chronotype and time-of-day influences on the alerting, orienting, and executive components of attention. Exp Brain Res. 2009 Jan;192(2):189-98. doi: 10.1007/s00221-008-1567-6. Epub 2008 Sep 23. Erratum in: Exp Brain Res. 2009 Jan;192(2):301. PMID: 18810396.

58. Walters AS, Silvestri R, Zucconi M, Chandrashekariah R, Konofal E. Review of the possible relationship and hypothetical links between attention deficit hyperactivity disorder (ADHD) and the simple sleep related movement disorders, parasomnias, hypersomnias, and circadian rhythm disorders. J Clin Sleep Med. 2008 Dec 15;4(6):591-600. PMID: 19110891; PMCID: PMC2603539.

59. Scheer FA, Shea TJ, Hilton MF, Shea SA. An endogenous circadian rhythm in sleep inertia results in greatest cognitive impairment upon awakening during the biological night. J Biol Rhythms. 2008 Aug;23(4):353-61. doi: 10.1177/0748730408318081. PMID: 18663242; PMCID: PMC3130065.

60. Allen PA, Grabbe J, McCarthy A, Bush AH, Wallace B. The early bird does not get the worm: time-of-day effects on college students' basic cognitive processing. Am J Psychol. 2008 Winter;121(4):551-64. PMID: 19105578.

61. Edwards B, Waterhouse J, Atkinson G, Reilly T. Effects of time of day and distance upon accuracy and consistency of throwing darts. J Sports Sci. 2007 Nov;25(13):1531-8. doi: 10.1080/02640410701244975. PMID: 17852679.

62. Edwards BJ, Lindsay K, Waterhouse J. Effect of time of day on the accuracy and consistency of the badminton serve. Ergonomics. 2005 Sep 15-Nov 15;48(11-14):1488-98. doi: 10.1080/00140130500100975. PMID: 16338715.

63. Van der Heijden KB, Smits MG, Van Someren EJ, Gunning WB. Idiopathic chronic sleep onset insomnia in attention-deficit/hyperactivity disorder: a circadian rhythm sleep disorder. Chronobiol Int. 2005;22(3):559-70. doi: 10.1081/CBI-200062410. PMID: 16076654.

64. Bonnefond A, Rohmer O, Hoeft A, Muzet A, Tassi P. Interaction of age with time of day and mental load in different cognitive tasks. Percept Mot Skills. 2003 Jun;96(3 Pt 2):1223-36. doi: 10.2466/pms.2003.96.3c.1223. PMID: 12929776.

65. Ohya Y, Ohtsubo T, Tsuchihashi T, Eto K, Sadanaga T, Nagao T, Abe I, Fujishima M. Altered diurnal variation of blood pressure in elderly subjects with decreased activity of daily living and impaired cognitive function. Hypertens Res. 2001 Nov;24(6):655-61. doi: 10.1291/hypres.24.655. PMID: 11768724

66. Sünram-Lea SI, Foster JK, Durlach P, Perez C. Glucose facilitation of cognitive performance in healthy young adults: examination of the influence of fast-duration, time of day and pre-consumption plasma glucose levels. Psychopharmacology (Berl). 2001 Aug;157(1):46-54. doi: 10.1007/s002130100771.

PMID: 11512042.

67. Kraemer S, Danker-Hopfe H, Dorn H, Schmidt A, Ehlert I, Herrmann WM. Time- of-day variations of indicators of attention: performance, physiologic parameters, and self-assessment of sleepiness. Biol Psychiatry. 2000 Dec 1;48(11):1069-80. doi: 10.1016/s0006-3223(00)00908-2. PMID: 11094140.

68. Liu Y, Higuchi S, Motohashi Y. Time-of-day effects of ethanol consumption on EEG topography and cognitive event-related potential in adult males. J Physiol Anthropol Appl Human Sci. 2000 Nov;19(6):249-54. doi: 10.2114/jpa.19.249. PMID:

11204871

69. Higuchi S, Liu Y, Yuasa T, Maeda A, Motohashi Y. Diurnal variation in the P300 component of human cognitive event-related potential. Chronobiol Int. 2000 Sep;17(5):669-78. doi: 10.1081/cbi-100101073. PMID: 11023214.

70. Yamadera H, Ito T, Suzuki H, Asayama K, Ito R, Endo S. Effects of bright light on cognitive and sleep-wake (circadian) rhythm disturbances in Alzheimer-type dementia. Psychiatry Clin Neurosci. 2000 Jun;54(3):352-3. doi: 10.1046/j.1440-1819.2000.00711.x. PMID: 11186110.

71. Winocur G, Hasher L. Aging and time-of-day effects on cognition in rats. Behav Neurosci. 1999 Oct;113(5):991-7. doi: 10.1037//0735-7044.113.5.991. PMID:

10571481

72. Brown LN, Goddard KM, Lahar CJ, Mosley JL. Age-related deficits in cognitive functioning are not mediated by time of day. Exp Aging Res. 1999 Jan- Mar;25(1):81-93. doi: 10.1080/036107399244156. PMID: 11370111.

73. Reinberg A, Bicakova-Rocher A, Nouguier J, Gorceix A, Mechkouri M, Touitou Y, Ashkenazi I. Circadian rhythm period in reaction time to light signals: difference between right- and left-hand side. Brain Res Cogn Brain Res. 1997 Oct;6(2):135-40. doi: 10.1016/s0926-6410(97)00024-4. PMID: 9450606.

74. Smith AP. Effects of time of day, introversion and neuroticism on selectivity in memory and attention. Percept Mot Skills. 1992 Jun;74(3 Pt 1):851-60. doi: 10.2466/pms.1992.74.3.851. PMID: 1608722.

75. Reinvang I, Bjartveit S, Johannessen SI, Hagen OP, Larsen S, Fagerthun H, Gjerstad L. Cognitive function and time-of-day variation in serum carbamazepine concentration in epileptic patients treated with monotherapy. Epilepsia. 1991

Jan-Feb;32(1):116-21. doi: 10.1111/j.1528-1157.1991.tb05621.x. PMID: 1985819.

76. van Lanschot JJ, Feenstra BW, Vermeij CG, Bruining HA. Accuracy of intermittent metabolic gas exchange recordings extrapolated for diurnal variation. Crit Care Med. 1988 Aug;16(8):737-42. doi: 10.1097/00003246-198808000-00001. PMID: 3396368.

77. Craig A, Condon R. Speed-accuracy trade-off and time of day. Acta Psychol (Amst). 1985 Feb;58(2):115-22. doi: 10.1016/0001-6918(85)90002-2. PMID: 3984775.

78. Jones BM. Circadian variation in the effects of alcohol on cognitive performance. Q J Stud Alcohol. 1974 Dec;35(4):1212-9. PMID: 4445461.

79. Mann H, Rutenfranz J, Stiller S. Untersuchungen zur Tagesperiodik der Reaktionszeit bei Nachtarbeit. IV. Tagesperiodische Anderungen der Parameter empirischer Reaktionszeitverteilungen [Circadian rhythm of reaction time during night work. IV. Diurnal variations in the parameters recorded for histograms of reaction times (author's transl)]. Int Arch Arbeitsmed. 1973 Jul 10;31(3):193-207. German. PMID: 4784736.

80. Mann H, Pöppel E, Rutenfranz J. Untersuchungen zur Tagesperiodik der Reaktionszeit bei Nachtarbeit. 3. Wechselbeziehungen zwischen Körpertemperatur und Reaktionszeit [Circadian rhythm of reaction time during night work. 3. Correlations between body temperature and reaction time]. Int Arch Arbeitsmed. 1972;29(4):269-84. German. PMID: 5049539.

81. Mann H, Rutenfranz J, Wever R. Untersuchungen zur Tagesperiodik der Reaktionszeit bei Nachtarbeit. II. Beziehungen zwischen Gleichwert und Schwingungsbreite [Circadian rhythm of reaction time during night work. II. Relation between mean and range of oscillation]. Int Arch Arbeitsmed. 1972;29(3):175-87. German. PMID: 5036155.

82. Mann H, Rutenfranz J, Aschoff J. Untersuchungen zur Tagesperiodik der Reaktionszeit bei Nachtarbeit. I. Die Phasenlage des positiven Scheitelwertes und Einflüsse des Schlafs auf die Schwingungsbrite [Circadian rhythm of reaction time during night work. I. Phase of maximum and influences of sleep on range of oscillation]. Int Arch Arbeitsmed. 1972;29(2):159-74. German. PMID: 5033355.

**Studies included in review:**

**N = 3**

61. Edwards B, Waterhouse J, Atkinson G, Reilly T. Effects of time of day and distance upon accuracy and consistency of throwing darts. J Sports Sci. 2007 Nov;25(13):1531-8. doi: 10.1080/02640410701244975. PMID: 17852679.

62. Edwards BJ, Lindsay K, Waterhouse J. Effect of time of day on the accuracy and consistency of the badminton serve. Ergonomics. 2005 Sep 15-Nov 15;48(11-14):1488-98. doi: 10.1080/00140130500100975. PMID: 16338715.

69. Higuchi S, Liu Y, Yuasa T, Maeda A, Motohashi Y. Diurnal variation in the P300 component of human cognitive event-related potential. Chronobiol Int. 2000 Sep;17(5):669-78. doi: 10.1081/cbi-100101073. PMID: 11023214.
